# Serial‐Passage Assessment Shows No Confirmed Resistance Development to Norway Spruce (*Picea Abies*) Resin in Bacterial Species Relevant to Wound Infection

**DOI:** 10.1002/mbo3.70436

**Published:** 2026-10-07

**Authors:** Kamilla Yamileva, Simone Parrotta, Maryam Ghanbarirad, Evgen Multia

**Affiliations:** ^1^ Repolar Pharmaceuticals Ltd. Espoo Finland; ^2^ Department of Bioproducts and Biosystems School of Chemical Engineering, Aalto University Espoo Finland

**Keywords:** antimicrobial resistance, Norway spruce resin, *Picea abies*, reduced susceptibility, serial passage, wound‐related bacteria

## Abstract

The search for antimicrobials with a low propensity to select resistance has intensified in response to the global antimicrobial resistance crisis. Norway spruce resin (*Picea abies*) has long been used in Northern European wound care traditions and has shown broad antimicrobial activity in earlier microbiological studies. In the present study, we evaluated whether prolonged exposure to medical‐grade spruce resin promotes reduced susceptibility in reference strains of three bacterial species relevant to wound infection. A 20‐day serial‐passage experiment was performed with *Staphylococcus aureus*, *Pseudomonas aeruginosa*, and *Enterococcus faecalis* using sub‐inhibitory resin concentrations and broth microdilution readouts at baseline, Day 10, and Day 20. Resistance development was predefined as a ≥ 4‐fold increase in inhibitory concentration. Baseline inhibitory concentrations were 1.25% for *S. aureus*, 5.0% for *P. aeruginosa*, and 2.5% for *E. faecalis*. After 20 days, inhibitory concentrations were 2.5%, 10.0%, and 2.5%, respectively, corresponding to at most twofold changes and remaining below the predefined threshold for resistance development. Validation and vehicle‐control arms indicated that these shifts were not attributable to medium transfer or solvent‐related bias. These findings suggest that medical‐grade Norway spruce resin has a low short‐term tendency to select for reduced susceptibility under serial‐passage conditions.

## Graphical abstract

Serial‐passage testing in wound‐relevant bacteria showed that Norway spruce (*Picea abies*) resin did not produce confirmed resistance development under the conditions tested. These findings support spruce resin as a natural antimicrobial with low propensity to select resistance.

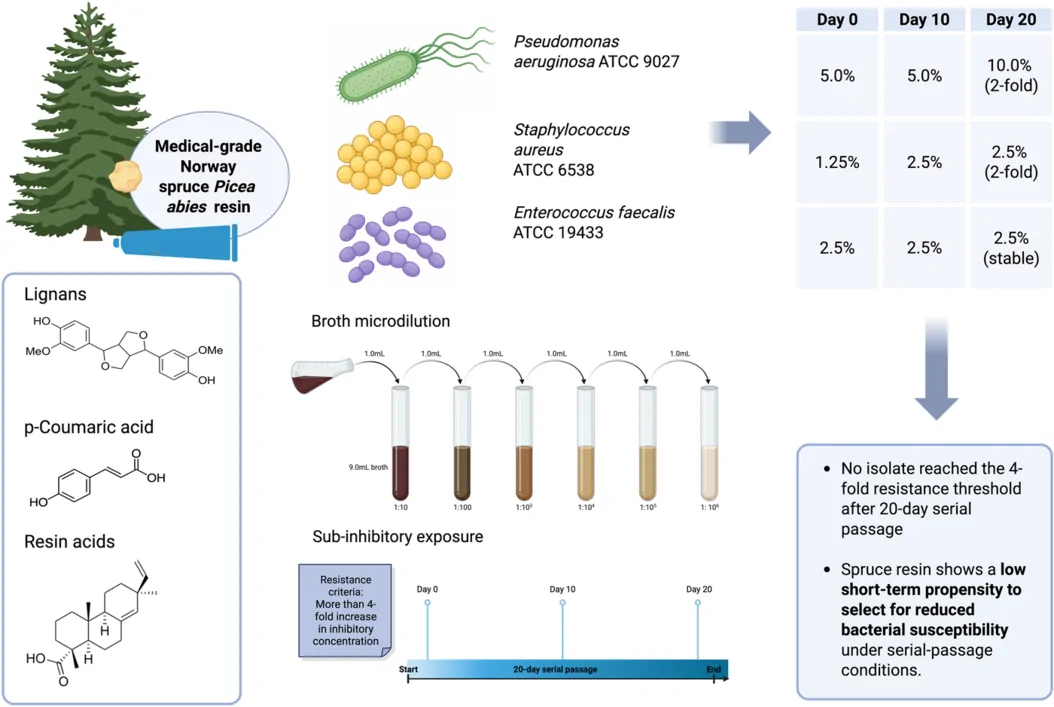

## 1 Introduction

Antimicrobial resistance has intensified the search for nonconventional antimicrobial agents that can control clinically relevant bacteria while exerting lower selective pressure for resistance than classical antibiotics (Chinemerem Nwobodo et al. 2022; Konwar et al. 2022). This need is particularly acute in wound care, where persistent microbial colonization, biofilm formation, and multidrug‐resistant pathogens can complicate treatment and delay healing (Metcalf and Bowler 2013). In some countries, topical antibiotic creams are still used in wound care (Fathy et al. 2021), but routine use is discouraged because clinical benefit is limited, prolonged exposure may favor resistant bacteria, and repeated application can cause contact allergy (Kreft and Wohlrab 2022). In this context, plant‐derived antimicrobials have attracted renewed interest because their activity often reflects chemically diverse phytoconstituents acting through multiple antibacterial mechanisms rather than a single defined target (Khameneh et al. 2019).

Among such natural antimicrobials, Norway spruce resin (*Picea abies*) has a distinctive position. In Northern Europe, salves prepared from spruce resin have been used in folk medicine for centuries to treat skin wounds, ulcers, and infections (Jokinen and Sipponen 2016). This long‐standing traditional use of spruce resin‐based wound products has inspired also modern laboratory (Goels et al. 2022; Goels et al. 2020; Eichenauer et al. 2023) and clinical investigation (Sipponen et al. 2012; Sipponen et al. 2008). Contemporary studies show that spruce resin and medical‐grade spruce resin preparations have antimicrobial activity (Rautio et al. 2007; Sipponen and Laitinen 2011; Sipponen et al. 2009; Jokinen and Sipponen 2016; Yamileva et al. 2025), wound‐healing potential (Sipponen et al. 2008; Sipponen et al. 2012), and favorable topical safety profile (Yamileva and Multia 2025; Jokinen and Sipponen 2016). In the earliest modern in vitro work, home‐made spruce resin salve showed bacteriostatic activity against clinically relevant wound‐associated bacteria, with the clearest effects against Gram‐positive organisms and demonstrable inhibition of methicillin‐resistant *Staphylococcus aureus* (MRSA) and vancomycin‐resistant enterococci (VRE) (Rautio et al. 2007). Later European Pharmacopoeia challenge‐test studies extended this picture by showing broad microbicidal activity of the resin against *S. aureus*, MRSA, *Escherichia coli*, *Pseudomonas aeruginosa*, *Klebsiella pneumoniae*, *Bacillus subtilis*, and *Candida albicans*, with 10% resin in the salve medium sufficient to prevent microbial survival under challenge‐test conditions (Sipponen and Laitinen 2011). Importantly, these studies also showed that apparent antimicrobial activity depends on assay format, since resin is poorly water‐soluble and diffusion‐based assays may therefore underestimate its effects, particularly against Gram‐negative bacteria and yeasts (Rautio et al. 2007; Sipponen and Laitinen 2011). In chemical terms, spruce resin is not a single active substance but a complex natural mixture in which resin acids predominate, together with lignans and *p*‐coumaric acid, a compositional profile that may help explain its broad biological activity (Sipponen and Laitinen 2011; Jokinen and Sipponen 2016). The antimicrobial activity of spruce resin likely arises from the combined effects of multiple constituent groups rather than from a single defined active compound, suggesting a mode of action that is broader and less target‐specific than that of conventional antibiotics.

Mechanistic evidence further suggests that spruce resin does not behave like a classical single‐target antibiotic. Ultrastructural and electrophysiological studies in *S. aureus* have shown cell‐wall thickening, cell aggregation, altered fatty‐acid composition, and dissipation of membrane potential after exposure to resin, resin salve, or abietic acid, indicating that antibacterial activity is associated with damage to the bacterial cell envelope and interference with energy metabolism (Sipponen et al. 2009). It has been proposed that terpenic resin acids are the main antimicrobial mediators, acting through nonspecific membrane‐ and cell wall‐associated effects, including disruption of the proton gradient and possible uncoupling of oxidative phosphorylation (Jokinen and Sipponen 2016). However, a recent study (Aimo‐Koivisto et al. 2024) of an aqueous spruce resin extract also showed marked ultrastructural damage in exposed microbes despite the absence of detectable non‐oxidized resin acids and the presence of only low levels of oxidized resin acids, lignans, and *p*‐coumaric acid, suggesting that antimicrobial activity may result from the combined effects of multiple resin‐derived constituents.

In the spruce resin literature, the emphasis has so far been on antimicrobial spectrum, assay‐dependent efficacy, ultrastructural mechanism, wound healing, biofilm eradication, and clinical use (Rautio et al. 2007; Sipponen and Laitinen 2011; Sipponen et al. 2009; Jokinen and Sipponen 2016; Junka et al. 2023; Hevér 2024). In a recent case report, continuous bacteriological sampling of the wound of one patient was performed, and despite targeted intravenous antibiotic treatment, *P. aeruginosa*, ESBL‐producing *K. pneumoniae*, *Morganella morganii*, and *E. faecalis* were cultured in the wound. One week after the use of spruce resin salve, it was observed that the pathogens cultured had changed to *Staphylococcus epidermidis*, *E. faecalis*, and *M. morganii* (Hevér 2024). More recent studies have further extended the evidence base by showing that spruce resin‐based salve can effectively eradicate mixed *P. aeruginosa*–*S. aureus* biofilm and penetrate its layers, even when the clinical isolates used exhibit high tolerance to bacitracin, neomycin, and polymyxin B (Junka et al. 2023). However, these studies have not directly examined whether repeated sub‐inhibitory exposure can select for reduced susceptibility. Thus, although spruce resin‐based preparations have been considered less likely than conventional antibiotics to promote resistance, this question has not yet been tested in a controlled serial‐passage experiment using medical‐grade spruce resin. This question is relevant to chronic wound care, where microbial populations can change over time under the influence of environmental contamination, host‐related selective pressures, and repeated antimicrobial treatment. Antibiotic exposure may favor less susceptible organisms, while biofilm growth can further increase tolerance and impair healing.

In the present study, we applied a serial‐passage approach to medical‐grade spruce resin used in resin salves such as Abilar, ilon Wundxtra Salbe, and SutriHeal Forte, to determine whether prolonged sub‐inhibitory exposure can drive reduced susceptibility. *S. aureus*, *P. aeruginosa*, and *E. faecalis* were selected as clinically important bacterial species relevant to wound infection and commonly associated with antimicrobial resistance and reduced treatment responsiveness (Guan et al. 2024; Pouget et al. 2022; Junka et al. 2023; Rautio et al. 2007). Accordingly, this study aimed to determine whether repeated exposure to medical‐grade Norway spruce resin makes bacteria less susceptible to its antimicrobial effects.

## 2 Materials and Methods

### 2.1 Study Design

A serial‐passage resistance study was conducted to evaluate whether prolonged exposure to spruce (*Picea abies*) resin could lead to reduced bacterial susceptibility. The resin was manually collected from sustainably managed forests in Finland, purified according to patented method (Sipponen 2011), and solubilized in ethanol. Serial dilutions were prepared in Mueller–Hinton broth (Biolife Cat No.: 4017412) to final test concentrations of 10.0%, 5.0%, 2.5%, 1.25%, 0.625%, 0.3125%, and 0.15625%.

### 2.2 Test Microorganisms and Culture Conditions

The organisms tested were *Staphylococcus aureus* ATCC 6538, *Pseudomonas aeruginosa* ATCC 9027, and *Enterococcus faecalis* ATCC 19433. Mueller–Hinton broth (MHB) was used for growth and Tryptic Soy Agar (TSA) (Neogen, Ref No.: NCM0004B) for plating. Incubation was carried out at 30°C–35°C for 18 h.

### 2.3 Serial‐Passage Protocol

This study was designed in accordance with CLSI guidelines for antimicrobial susceptibility testing (M07‐A9: Methods for Dilution Antimicrobial Susceptibility Tests for Bacteria That Grow Aerobically) and adapted for resistance evaluation through serial passage. In this experimental setup, the test product was introduced at sub‐inhibitory concentrations (typically 0.25× to 0.5× of the initial minimum inhibitory concentration [MIC]) into liquid culture medium. The selected test microorganism was inoculated and incubated under optimal growth conditions. After each incubation cycle, cultures were transferred daily into fresh medium containing the same sub‐inhibitory concentration of the product, for a predefined number of passages (10 and 20 passages). MIC determinations were performed at baseline and at scheduled intervals (midpoint of 10 days and final passage after 20 days) using broth microdilution methodology. MIC results were evaluated descriptively as fold changes relative to baseline. Resistance development was predefined as a ≥ 4‐fold increase in MIC. Inferential statistical testing was not performed because MIC values were discrete endpoints obtained from a twofold dilution series, only duplicate determinations were conducted, and the duplicate trials yielded identical MIC endpoints. This approach allowed monitoring of adaptive changes in susceptibility under prolonged exposure conditions. All assays were performed in duplicate. Since ethanol was used as solvent, vehicle controls containing ethanol without active resin were included. Validation passages without product exposure and validation MIC determinations from nutrient broth subcultures were also performed to exclude adaptation to laboratory conditions or medium transfer artefacts.

## 3 Results

### 3.1 Baseline Inhibitory Concentrations

At baseline (Day 0, Table 1), medical‐grade spruce resin inhibited *P. aeruginosa* at 5.0%, *S. aureus* at 1.25%, and *E. faecalis* at 2.5%. Duplicate determinations yielded identical MIC endpoints at all assessed time points. Therefore, one combined summary table is presented, and the results are reported descriptively as observed MIC values and fold changes relative to baseline. These values indicate that *S. aureus* was the most susceptible of the three tested organisms, while *P. aeruginosa* required the highest concentration.

**Table 1:** Summary of broth microdilution growth patterns for spruce resin during serial passage at Days 0, 10, and 20. All assays were performed in duplicate.

Test organism	Time point	10%	5%	2.5%	1.25%	0.625%	0.3125%	0.15625%	PC	NC
*P. aeruginosa*	Day 0	NG	**NG**	G	G	G	G	G	G	NG
*P. aeruginosa*	Day 10	NG	**NG**	G	G	G	G	G	G	NG
*P. aeruginosa*	Day 20	**NG**	G	G	G	G	G	G	G	NG
*S. aureus*	Day 0	NG	NG	NG	**NG**	G	G	G	G	NG
*S. aureus*	Day 10	NG	NG	**NG**	G	G	G	G	G	NG
*S. aureus*	Day 20	NG	NG	**NG**	G	G	G	G	G	NG
*E. faecalis*	Day 0	NG	NG	**NG**	G	G	G	G	G	NG
*E. faecalis*	Day 10	NG	NG	**NG**	G	G	G	G	G	NG
*E. faecalis*	Day 20	NG	NG	**NG**	G	G	G	G	G	NG

*Note:* MIC endpoint was defined as the lowest resin concentration showing no visible growth. Resistance development was predefined as a ≥ 4‐fold increase in MIC relative to baseline. Because the concentrations are presented in descending order, growth at 5.0% and no growth at 10.0% for *P. aeruginosa* at Day 20 correspond to an MIC of 10.0%. MIC endpoints are shown in bold.

Abbreviations: G, growth observed; NC, negative control; NG, no growth observed; PC, positive control.

### 3.2 Serial‐Passage Susceptibility Over 20 Days

Across 20 days of serial passage, susceptibility remained broadly stable. For *P. aeruginosa*, the inhibitory concentration remained 5.0% at Day 10 and increased to 10.0% at Day 20, representing a twofold increase from the baseline MIC of 5.0%. For *S. aureus*, the inhibitory concentration increased from 1.25% at baseline to 2.5% at Day 10 and remained 2.5% at Day 20, also a twofold change. For *E. faecalis*, the inhibitory concentration remained unchanged at 2.5% throughout the experiment.

Vehicle‐control and validation arms (Table 2) remained stable over time and did not show progressive increases suggestive of methodological drift. The transfer procedure and medium changes did not appear to induce artificial resistance. Vehicle‐control and validation endpoints remained stable over time and showed no progressive increases suggestive of methodological drift. The Day‐20 increase seen in *P. aeruginosa* under test conditions was not mirrored in the validation arms. None of the tested bacteria showed a ≥ 4‐fold increase in inhibitory concentration, and thus none met the predefined criterion for resistance development. The observed changes were limited to minor twofold shifts in *P. aeruginosa* and *S. aureus* (Table 2 and Figure 1).

**Table 2:** Inhibitory concentrations in resin‐exposed, matched ethanol vehicle‐control, and validation arms during the 20‐day serial‐passage study.

Test organism	Condition	Day 0	Day 10	Day 20
*P. aeruginosa*	Resin‐exposed	5.00%	5.00%	10.00%
	Vehicle control	5.00%	5.00%	5.00%
	Validation: MHB to resin	n/a	5.00%	5.00%
	Validation: MHB to ethanol	n/a	5.00%	2.50%
*S. aureus*	Resin‐exposed	1.25%	2.50%	2.50%
	Vehicle control	10.00%	10.00%	10.00%
	Validation: MHB to resin	n/a	2.50%	1.25%
	Validation: MHB to ethanol	n/a	10.00%	10.00%
*E. faecalis*	Resin‐exposed	2.50%	2.50%	2.50%
	Vehicle control	10.00%	10.00%	10.00%
	Validation: MHB to resin	n/a	2.50%	2.50%
	Validation: MHB to ethanol	n/a	10.00%	10.00%

*Note:* Resin‐exposed cultures were serially passaged at sub‐inhibitory resin concentrations. Vehicle‐control cultures were serially passaged in ethanol without resin. Validation cultures were passaged in Mueller–Hinton broth (MHB) without resin exposure and subsequently tested against resin or ethanol.

Abbreviation: n/a = not applicable.

**Figure 1:**
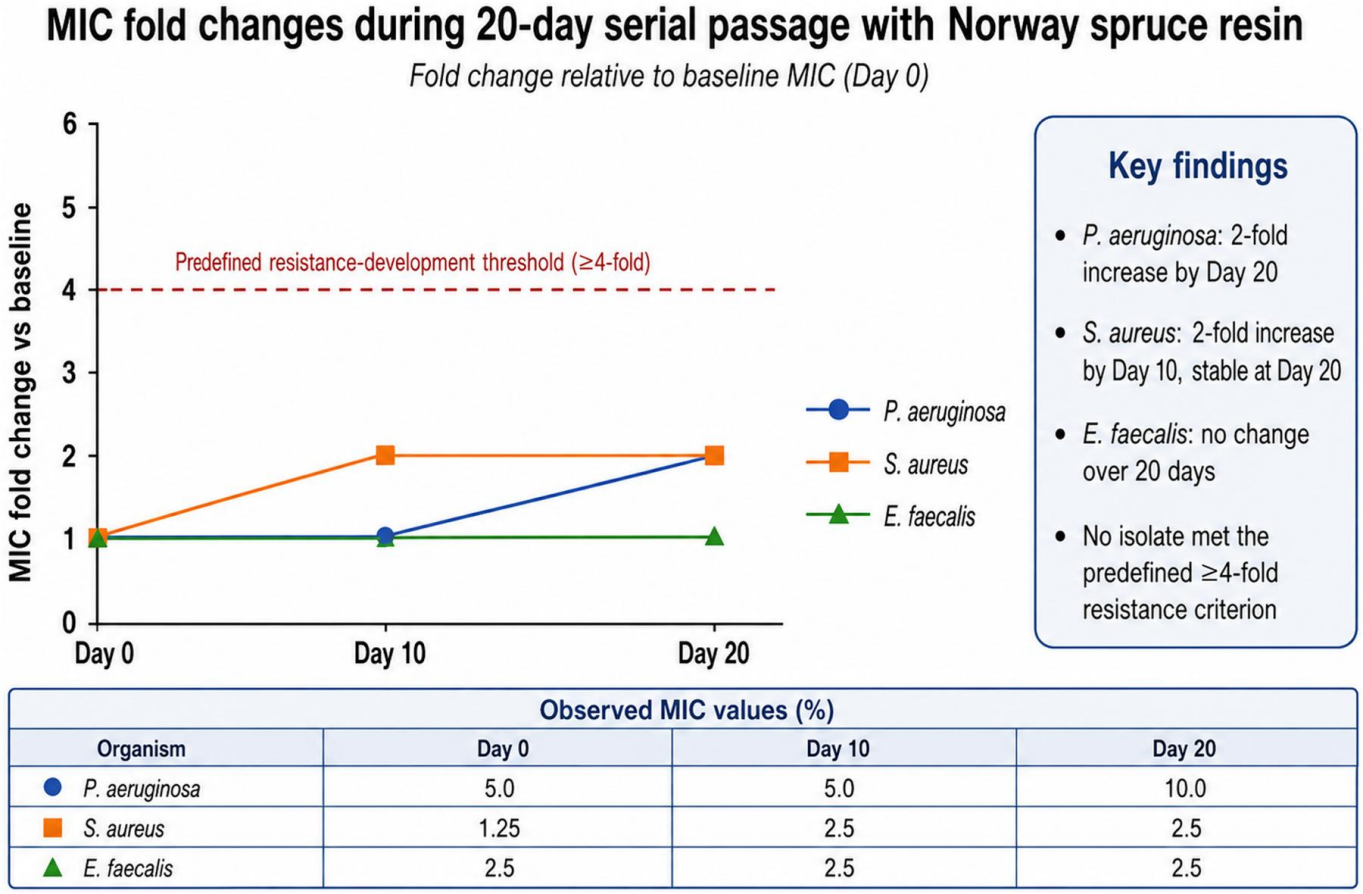
Fold changes in medical‐grade spruce resin minimum inhibitory concentration during the 20‐day serial‐passage experiment. MIC values at Days 10 and 20 were normalized to the corresponding baseline MIC for each bacterial species. The horizontal dashed line indicates the predefined fourfold increase used as the criterion for potential resistance development. Duplicate determinations produced identical MIC endpoints.

20 days of serial passages were used to assess short‐term resistance selection potential because repeated sub‐inhibitory exposure over many consecutive transfers gives bacterial populations sustained opportunity to adapt (Tambe et al. 2001). However, the 20‐day design cannot exclude slower adaptive responses that may emerge during longer‐term exposure. Overall, the susceptibility profiles remained stable, with only minor twofold variations in two organisms and no measurable change in the third.

To facilitate comparison between organisms and time points, MIC values were also expressed as fold changes relative to baseline (Figure 1). The largest observed change was twofold for *P. aeruginosa* and *S. aureus*, whereas the MIC for *E. faecalis* remained unchanged. All values remained below the predefined fourfold threshold for potential resistance development.

## 4 Discussion

In this study, spruce resin showed a low tendency to select for reduced bacterial susceptibility during 20 days of serial passage in three clinically important wound‐associated bacteria. None of the tested organisms reached the predefined resistance threshold of a ≥ 4‐fold increase in MIC. Instead, the observed changes were limited to a twofold increase in *P. aeruginosa* and *S. aureus*, while *E. faecalis* remained unchanged throughout the experiment. These findings suggest that, under the present test conditions, repeated sub‐inhibitory exposure to medical‐grade spruce resin did not produce a phenotypically detectable resistance‐level increase in MIC. This has practical relevance for using spruce resin for infected wounds, where microorganisms may encounter repeated topical treatment under variable local exposure conditions, including sub‐inhibitory levels at the wound surface or biofilm interface.

In serial‐passage experiments, small changes, e.g., twofold shifts of *P. aeruginosa* and *S. aureus* do not necessarily indicate stable resistance (Kadeřábková et al. 2024; Rosenblatt et al. 2019), especially when they remain below a predefined biological threshold and are not supported by consistent drift in validation or vehicle‐control arms. The inclusion of ethanol controls and validation passages helped to exclude medium transfer effects, solvent‐related bias, or simple adaptation to laboratory passage conditions. For *S. aureus* and *E. faecalis*, the vehicle‐control inhibitory endpoint remained at 10.0% throughout the experiment, whereas the corresponding resin‐containing test endpoints were lower. This indicates that the inhibitory activity observed at lower test concentrations cannot be explained by ethanol alone, although ethanol may influence resin solubility, bacterial physiology, or the absolute inhibitory endpoint. Importantly, the vehicle‐control values remained stable over time and showed no progressive shift during serial passage. Similarly, the Day 20 increase observed for *P. aeruginosa* under resin exposure was not reproduced in the validation arms, supporting the interpretation that the change was minor and not clearly indicative of progressive resistance selection.

Although closely comparable studies are scarce, published serial‐passage experiments with conventional antibiotics provide useful context (D'Lima et al. 2012). In MRSA, 50 serial passages to topical comparators such as mupirocin (64‐fold increase), fusidic acid (256‐fold increase), and retapamulin (16‐fold increase) has produced MIC increases (D'Lima et al. 2012) well above the fourfold threshold used in this study, and ciprofloxacin has similarly generated 32‐fold MIC increases in both *S. aureus* and *P. aeruginosa* after 25 serial passages. Serial‐passage selection has also been demonstrated in *E. faecalis* with linezolid (Lobritz et al. 2003). These findings contrast with the twofold shifts observed for spruce resin in the present study, although differences in experimental design limit direct comparability.

Phenotypic MIC measurements cannot exclude genetic adaptation during repeated antimicrobial exposure. Although the ≥ 4‐fold increase used here provides an operational criterion for resistance development, adaptive genomic changes may occur before substantial MIC shifts become detectable. This is relevant to spruce resin, whose antibacterial activity appears to involve multiple constituents and effects on the bacterial cell envelope rather than a single defined target. Thus, the absence of a ≥ 4‐fold MIC increase indicates no phenotypically detectable resistance under the conditions tested, but does not exclude underlying genetic adaptation. Whole‐genome sequencing (Wang 2025; Pei 2026; Jia et al. 2025) has become an important complement to phenotypic susceptibility testing because it can identify genetic changes associated with antimicrobial adaptation that may not be reflected by substantial changes in MIC. In future serial‐passage studies of spruce resin, sequencing of baseline and endpoint isolates could therefore determine whether repeated exposure selects genetic changes in wound‐associated bacteria despite phenotypically stable susceptibility. Such analyses could be complemented by fitness and cross‐resistance assessments. Study results extend earlier evidence of broad antimicrobial activity by addressing a question that previous spruce‐resin studies had not directly examined, namely resistance selection under repeated exposure. Previous studies (Rautio et al. 2007) demonstrated activity against Gram‐positive wound‐associated bacteria, including MRSA and VRE, and later challenge‐test studies extended this evidence to Gram‐negative organisms and *Candida albicans* (Sipponen and Laitinen 2011). Additional mechanistic studies suggested that spruce resin acts through relatively nonspecific effects on the bacterial cell envelope, including cell wall and membrane damage, altered fatty‐acid composition, and dissipation of membrane potential (Sipponen et al. 2009). Such a mode of action differs from that of classical single‐target antibiotics (Silver 2011) and may partly explain why marked resistance selection was not observed in the present serial‐passage model. Differences between species were also biologically plausible. *S. aureus* was the most susceptible organism at baseline, whereas *P. aeruginosa* required the highest inhibitory concentration, consistent with the greater intrinsic tolerance often seen in Gram‐negative bacteria and with earlier observations that apparent resin activity may depend on assay format (Rautio et al. 2007; Sipponen and Laitinen 2011; Sipponen 2013). *E. faecalis* remained stable during passage. The results suggest that any adaptive response to spruce resin, if present, was limited and species‐dependent under the tested conditions.

Although previous studies have reported cell‐wall and membrane alterations, changes in fatty‐acid composition, dissipation of membrane potential, and ultrastructural damage following exposure to spruce resin or its constituents, the present study did not directly investigate these mechanisms. Future studies should examine isolates collected during extended serial passage using microscopy, membrane‐integrity and permeability assays, membrane‐potential measurements, and molecular analyses to determine whether cellular adaptations occur even in the absence of substantial MIC changes.

From a clinical and translational perspective, these findings strengthen the rationale for continued use of spruce resin as a topical antimicrobial for wound management. Microorganisms in chronic wounds may encounter variable and sometimes sub‐inhibitory exposure to topical antimicrobials because wound exudate can reduce bactericidal efficacy under wound‐bed conditions (Radischat et al. 2020), while biofilms increase tolerance and impair antimicrobial effectiveness (Percival et al. 2011; Omar et al. 2017). Biofilm growth may also alter resistance‐selection dynamics compared with planktonic serial passage. This is relevant to spruce resin because antimicrobial activity has previously been demonstrated against mixed *P. aeruginosa–S. aureus* biofilms (Junka et al. 2023), whereas the effect of repeated sub‐inhibitory exposure under biofilm conditions remains unknown. Future studies should therefore assess resistance selection in biofilm‐based models that more closely reflect chronic wound environments. Overall, the present findings indicate a low short‐term tendency of spruce resin to select for reduced susceptibility under the conditions tested.

The 20‐day serial‐passage design does not exclude slower adaptive responses that might emerge during more prolonged exposure. Longer‐term experiments, including studies extending to 50 or more passages as well as continuous‐flow and biofilm‐based models, are therefore needed to determine whether such responses occur and whether the observed susceptibility profile is maintained across diverse and clinically resistant strains. In addition, this study assessed only phenotypic changes in inhibitory concentration and did not examine stable genetic adaptation, transcriptomic responses, fitness costs, cross‐resistance to conventional antibiotics, or biofilm‐related changes. Future studies should therefore combine extended exposure models with whole‐genome sequencing or transcriptomic analyses, bacterial fitness measurements, and susceptibility testing against other antimicrobial agents. The use of well‐characterized reference strains enabled a controlled initial assessment but limits the clinical generalizability of the findings. Clinical isolates may harbor pre‐existing resistance determinants and adaptive traits that could influence their response to repeated resin exposure and the emergence of reduced susceptibility. Future studies should therefore include genetically and phenotypically characterized clinical isolates, particularly MRSA, VRE‐associated enterococci, and multidrug‐resistant *P. aeruginosa*.

## 5 Conclusion

Norway spruce (*Picea abies*) resin maintained antibacterial activity throughout a 20‐day serial‐passage experiment against *P. aeruginosa*, *S. aureus*, and *E. faecalis*. None of the tested organisms showed the predefined ≥ 4‐fold increase in inhibitory concentration required to indicate resistance development. These findings suggest that, under the conditions tested, medical‐grade spruce resin has a low short‐term tendency to select for reduced susceptibility. Longer‐term studies, including experiments extending to 50 or more serial passages, continuous‐flow systems, and biofilm‐based models, are needed to determine whether slower adaptive responses emerge and whether the observed susceptibility profile is maintained across diverse clinical and antimicrobial‐resistant isolates.

## Author Contributions

**Kamilla Yamileva:** writing – original draft, writing – review and editing, visualization. **Simone Parrotta:** writing – review and editing, methodology, investigation. **Maryam Ghanbarirad:** writing – review and editing, resources, funding acquisition, project administration. **Evgen Multia:** writing – original draft, writing – review and editing, visualization, supervision, resources, project administration, methodology, investigation, funding acquisition, formal analysis, data curation, conceptualization.

## Funding

The authors have nothing to report.

## Ethics Statement

The authors have nothing to report.

## Conflicts of Interest

Kamilla Yamileva and Maryam Ghanbarirad have employment at Repolar Pharmaceuticals Ltd.

## Data Availability

All data generated or analyzed during this study are included in this published article.
